# An Update on the Status of Potent Inhibitors of Metallo-β-Lactamases

**DOI:** 10.3797/scipharm.1302-08

**Published:** 2013-03-28

**Authors:** Faridoon, Nazar Ul Islam

**Affiliations:** 1 Chemistry Department, Islamia College University, Peshawar-25120, Pakistan.; 2 Institute of Chemical Sciences, University of Peshawar, Peshawar-25120, Pakistan.

**Keywords:** Metallo-beta-lactamases, Inhibition, Thiol, β-Lactam analogues, Peptides, Biphenyl tetrazoles, Triazoles

## Abstract

The production of metallo-β-lactamases is the most important strategy by which pathogenic bacteria become resistant to currently known β-lactam antibiotics. The emergence of these enzymes is particularly concerning for the future treatment of bacterial infections. There are no clinically available drugs capable of inhibiting any of the metallo-β-lactamases, so there is an urgent need to find such inhibitors. In this review, an up-to-date status of the inhibitors investigated for the inhibition of metallo-β-lactamases has been given so that this rich source of structural information of presently known metallo-β-lactamases could be helpful in generating a broad-spectrum potent inhibitor of metallo-β-lactamases.

## Introduction

β-Lactam antibiotics are the most widely used antibacterial agents for treating bacterial infections. However, many pathogenic bacteria have developed resistance against these antibiotics through mechanisms such as a decrease in cell wall permeability, efflux pump, and hydrolysis of the β-lactam ring by β-lactamases [1, 2]. β-Lactamases of class B are metallo-proteins, also called metallo-β-lactamases. These enzymes use a zinc-bound hydroxyl group as the nucleophile [3] to promote the hydrolysis of a very broad range of β-lactam antibiotics, including penicillins, cephalosporins, and carbapenems [4].

Serine-β-lactamase inhibitors do not inhibit metallo-β-lactamases and there are no clinically approved inhibitors of metallo-β-lactamases. So there is an urgent need for the development of such an inhibitor that could inhibit all metallo-β-lactamases. Here, we discuss currently available MBL inhibitors from different groups to provide a collective view of the chemical structures of these inhibitors, which could be helpful in designing inhibitors capable of meeting the serious biological threat, resistance of pathogenic bacteria to β-lactam antibiotics.

## Phenazines

**Fig. 1:**
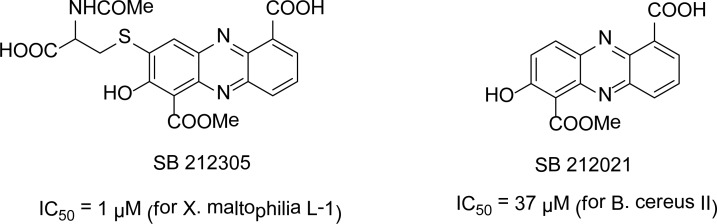
Phenazine inhibitors

Gilpin *et al*., [5] isolated two novel phenazines (SB 212021 and SB212305) from a *streptomyces* in 1995 and screened them against three metallo-β-lactamases *Xanthomonas maltophilia* L-l, *Bacteroides fragilis* 262 CfiA, and *Bacillus cereus* II. These compounds are non-specific and appear to chelate the zinc, so when zinc levels are increased, enzyme activity recovers. In the absence of added zinc, both compounds had IC_50_s of 1∼75 μM for the *B. fragilis* 262 CfiA and *X. maltophilia* L-1 metallo-β-lactamases. SB212305, which contains a thio group, has the lowest IC_50_ = 1 μM for *X. maltophilia* L-1, while in the case of *B. cereus* II, the phenazine SB212021 has the lowest IC_50_ = 37 μM (Figure 1).

## Thiol derivatives

Due to the high affinity of sulfur for zinc, it has been found that compounds having a thiol group showed promising inhibition against MBLs. A series of mercaptoacetic acid thiol esters [6] was synthesized and identified as metallo-β-lactamase inhibitors. Mass spectrometric data suggest that mercaptoacetic thiol esters act as mechanism-based inhibitors for BcII by generating mercaptoacetic acid *in situ,* which forms a disulfide linkage with a cysteine residue in the active site of the enzyme. The inhibitors of this series showed a broad range of potencies (IC_50_’s varied from mM to μM) against the enzymes.

**Fig. 2:**
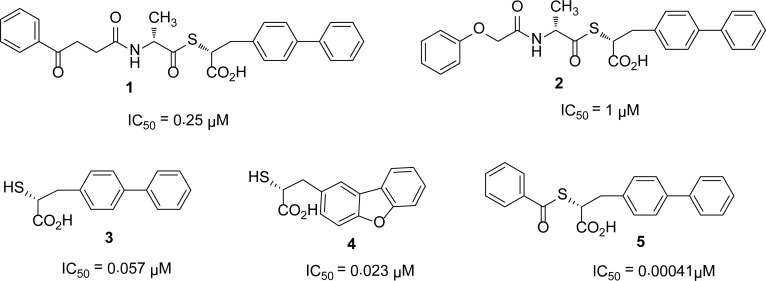
Comparison of IC_50_ values of thiols and thiol esters

A series of thioesters and thiols synthesized by a novel solid-phase Mitsunobu reaction were screened [7] against the CcrA and IMP-1 enzymes. These compounds showed better inhibition for IMP-1 than for CcrA MBL. In the thiodepsipeptides series, the most potent inhibitor was compound **1** with an IC_50_ of 0.25 μM against IMP-1. However, the thiol moieties of the thiodepsipeptides showed better inhibition, with IC_50_s of 0.086–0.023 μM against IMP-1. On the other hand, the simple acetyl and benzoyl thioesters were found to be significantly more potent inhibitors than the thiols themselves. This comparison of activity of thiols and thioesters is shown in the Figure 2.

In 1998, S. Bounaga and his co-workers [8] reported *N*-(2′-mercaptoethyl)-2-phenylacetamide **6** as a competitive inhibitor of β-lactamase II from *B. cereus* with a *K*_i_ of 70 mM. This compound was also identified as a competitive inhibitor of L1 with a *K*_i_ of 50 ± 3 μM [9].

**Fig. 3:**
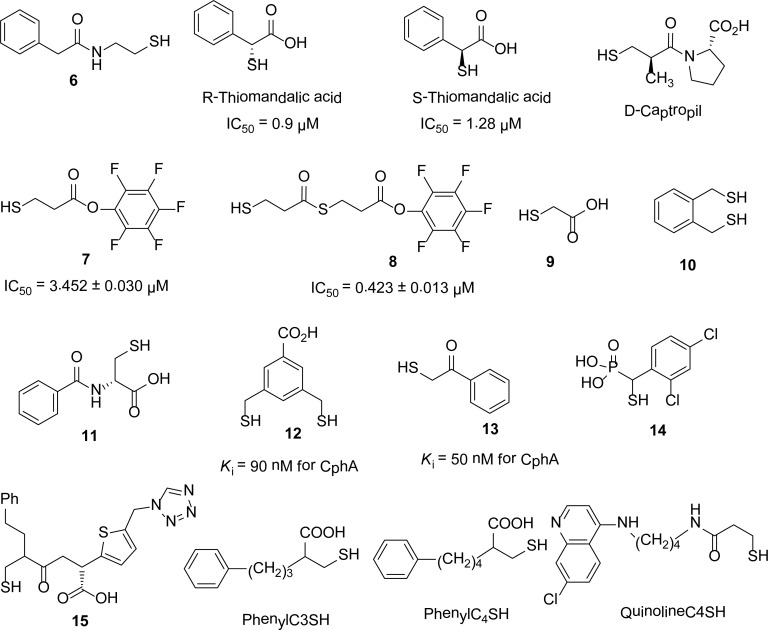
Thiols inhibitors of MBLs

Thiomandelic acid [10] was identified as a broad spectrum inhibitor with a sub-micromolar *K_i_* value for the nine MBLs tested, except that from the *Aeromonas hydrophila* enzyme. The *K_i_* values of *R* and *S*-thiomandelic acids for *B. cereus* enzyme were found 0.09 and 1.28 μM, respectively (Figure 3). Kurosaki *at al*., [11] reported the two irreversible inhibitors **7** and **8** with *K_i_* values of 3.452 ± 0.030 μM and 0.423 ± 0.013 μM, respectively for the IMP-1 enzyme (Figure 3).

Siemann *et al*., also studied thiols as classical inhibitors of MBLs and reported mercaptoacetic acid **9** (IC_50_ = 1.3 μM) and 1,2-benzenedimethanethiol **10** (IC_50_ = 2.3 μM) as inhibitors of the IMP-1 enzyme [12]. Demonstrating the efficiency of the Dynamic Combinatorial Mass Spectrometry (DCMS) technique, *N*-Benzoyl-D-cysteine **11**[13] was identified as the potent inhibitor for BcII enzyme with a *K*_i_ value of 740 nM. D-captopril [14] was reported as a broad spectrum potent inhibitor of subclass B1 and B3, but its potency against subclass B2 is low (*K_i_* = 72 μM).

In search of potent inhibitors of all subclasses of MBLs, Liénard and his co-workers [15] synthesized compounds containing thiol function(s). Compounds **12** and **13** were found to inhibit MBLs from all three subclasses. For the monozinc CphA MBL, compounds **12** and **13** were reported to be the most potent inhibitors with *K*_i_ = 90 nM and *K*_i_ = 50 nM, respectively. Lassaux *et al*., [16] synthesized mercaptophosphonate compounds and screened them against all subclasses of MBLs. Most of their synthesized compounds were found to be competitive inhibitors for all three subclasses B1, B2, and B3 MBLs. With some exceptions, all the mercaptophosphonate derivatives showed good inhibitory effects on the CphA, a subclass B2 enzyme with low inhibition constants (*K_i_* < 15 μM). The most potent broad spectrum inhibitor **14** of this series is given in Figure 3.

The mercaptocarboxylate, PhenylC3SH (Figure 3) [17], acts as a potent inhibitor of the VIM-2 enzyme with a *K*_i_ value of 220 nM, whereas it is less active for the IMP-1 enzyme (*K*_i_ = 1660 nM) [18].

Concha and others [19] determined the crystal structure of the IMP-1- mercaptocarboxylate **15** complex and found that this mercaptocarboxylate inhibits the IMP-1, *B. fragilis*, and L1 enzymes with IC_50_ values between 100 and 500 nM. W. Jin [18] and his colleagues studied the inhibitory effect of two series of compounds, 2-ω-phenylalkyl-3-mercaptopropionic acids and N-[(7-chloro-quinolin-4-ylamino)-alkyl]-3-mercapto-propionamides, on IMP-1 and VIM-2 metallo-β-lactamases. Among the first series, PhenylC4SH (Figure 3) was found to be the potent inhibitor of both IMP-1 and VIM-2 with IC_50_ values of 1.2 and 1.1 μM, respectively, whereas QuinolineC4SH (Figure 3) of the second series showed maximum inhibition for the IMP-1 (IC_50_ = 2.5 μM) and VIM-2 (IC_50_ = 2.4 μM) enzymes.

## Trifluoromethyl alcohols and ketones

**Fig. 4:**
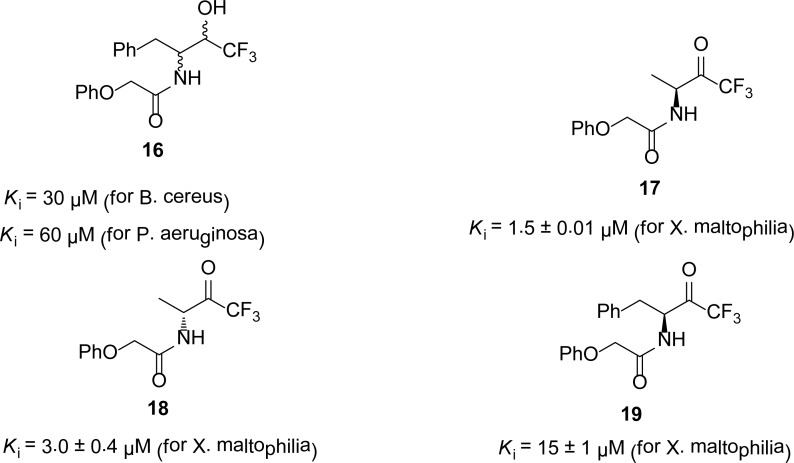
*K*_i_ values of trifluoromethyl ketones and alcohol

Trifluoromethyl ketones are reported as serine proteases [20, 21] and zinc dependent carboxypeptidase [22] enzymes. Walter *et al*., [23, 24] reported the trifluoromethyl compounds as the first synthetic inhibitors of different strains of MBLs. They synthesized different *N*-phenoxyacetyl-substituted trifluoromethyl ketones and alcohols and screened against MBLs from *X. maltophilia* ULA-511, *A. hydrophila* AE036, *B. cereus* 569H, and *Pseudomonas aeruginosa* 101 as inhibitors. Among these trifluoromethyl ketones and alcohols, the most potent inhibitors **16–19** are given in Figure 4 along with the *K*_i_ values.

## Biphenyl tetrazoles

**Fig. 5:**
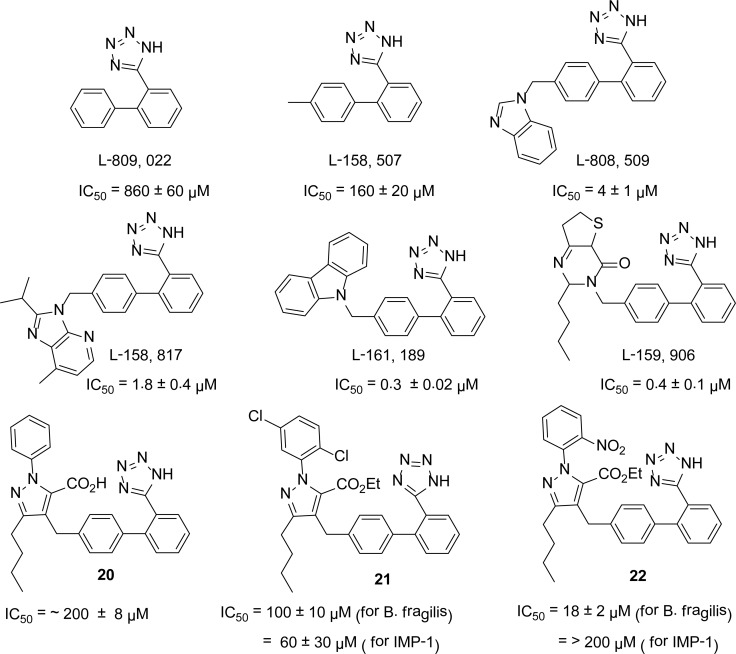
Comparison of IC_50_ values of BPT and substituted BPTs

Screening of the Merck chemical collection by Toney *et al*., [25] led to the identification of biphenyl tetrazoles as potent competitive inhibitors of metallo-β-lactamase (*B. fragilis*). The structure-activity relationships of biphenyl tetrazoles showed that unsubstituted BPT is a poor inhibitor with IC_50_ value of 860 ± 60 μM. It was found that the ortho position of the tetrazole group, relative to the biphenyl ring system, is important in enzyme inhibition, because movement of the tetrazole group to the meta or para positions led to the loss of inhibitory activity [25]. It was also found that substitution at the 4′-position of the BPT further increased the inhibitory activity of the BPTs. Figure 5 shows the IC_50_ values comparison of the parent BPT and the substituted BPTs. To further explore the potency of BPTs as MBLs inhibitors, in 1999, Toney *et al*., [26] screened a series of BPTs containing 3-*n*-butyl-1-phenylpyrazole-5-carboxylate against *B. fragilis* and IMP-1 metallo-β-lactamases. The parent BPT **20** was found to be a weak inhibitor with an IC_50_ of ∼ 200 ± 8 μM of *B. fragilis* MBL, while against IMP-1, it was found to be inactive (IC_50_ >200 μM). The substitution upon the phenyl ring and esterification of the carboxylic group of compound **20** lowered the IC_50_ value up to 60 ± 30 μM **21** for IMP-1 and 18 ± 2 μM **22** for *B. fragilis* MBL (Figure 5).

## Succinic and phthalic acid derivatives

The IMP-1 enzyme is a plasmid-borne zinc metalloenzyme responsible for the hydrolysis of β-lactam antibiotics, including carbapenems, rendering them ineffective. To protect the broad spectrum antibiotics from hydrolysis by IMP-1, Toney *et al*., [27] have identified a series of 2,3-(*S,S*)-disubstituted succinic acids as potent inhibitors of IMP-1. Among this series, the most potent inhibitor is **23** with an IC_50_ of 0.0027 μM. In 2005, Toney [28] and others reported several novel succinic acid derivatives, as IMP-1 inhibitors showing mixed inhibition. They reported compound **20707** with lowest *K*_i_ value of 3.3 ± 1.7 μM.

**Fig. 6:**
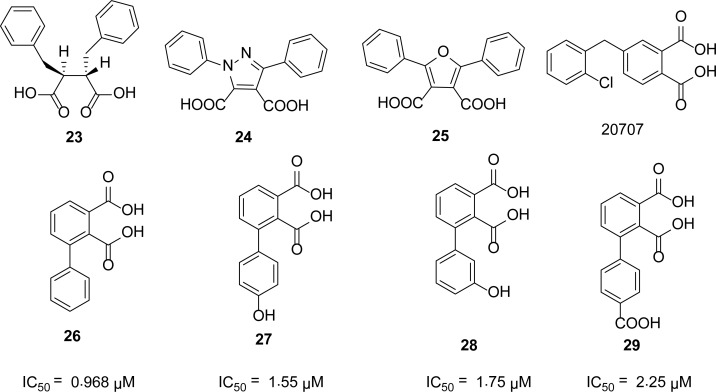
Succinic and phthalic acid derivatives as MBL inhibitors

Using docking methodologies and experimental enzyme kinetics, Olsen [29] and his coworkers identified several succinic acid derivatives as the di-zinc metallo-β-lactamase inhibitors. The potent inhibitors, **24** and **25** (Figure 6), of this series have an IC_50_ value range of 10-100 μM. Hiraiwa *et al*., [30] synthesized substituted phthalic acids and screened against the IMP-1 enzyme for inhibitory activity. Phthalic acid has almost no inhibitory activity against IMP-1, but 3-substituted phthalic acid derivatives are potent inhibitors of this enzyme. Some of the most potent inhibitors **26–29** of this series, along with their IC_50_ values, are given in Figure 6.

## Hydroxamates

**Fig. 7:**
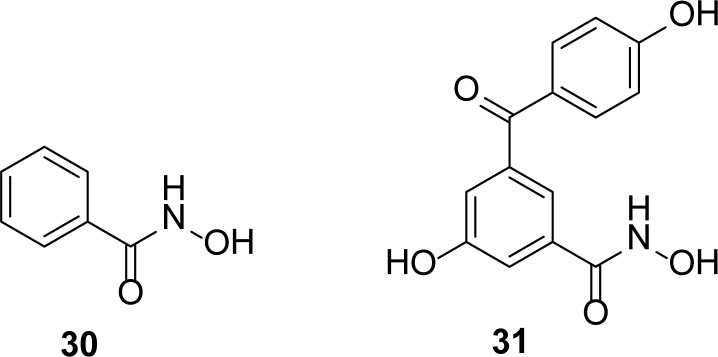
Hydroxamates as FEZ-1 inhibitor

Walter *et al*., [31] synthesized amino acid-derived hydroxamates and screened against different MBLs for inhibitory activity and found several compounds as the inhibitors of clinically relevant enzymes from *A. hydrophila*. In 2006, B. M. R. Liénard and his colleagues [32] synthesized a series of derivatives of benzohydroxamic acid **30** and tested them against FEZ-1, IMP-1, BcII, CphA, and L1 MBLs for inhibitory activity. Their study resulted in the identification of selective inhibitors of FEZ-1 metallo-β-lactamase. The most potent selective inhibitor of FEZ-1 from this series was identified as 2,5-substituted benzophenone hydroxamic acid **31** (Figure 7) with a *K_i_* value of 6.1 ± 0.7 μM.

## β-Lactam analogues

In search of broad spectrum potent inhibitors of MBLs, a variety of 1β-methylcarbapenem conjugates were tested against different MBLs [33]. The kinetic studies showed that 1β-methylcarbapenems having dithiocarbamate, benzothienythio, or pyrrolidinylthio moieties at the C-2 position showed promising inhibition against MBLs. The most potent inhibitor among these compounds is J-110,441, which simultaneously targets class A, B, and C β-lactamases. It almost acts as a broad spectrum inhibitor of MBLs with *K*_i_ values of 0.83, 1.00, 0.23, and 0.0037 μM for II from *B. cereus*, L1from *Stenotrophomonas maltophilia*, CcrA from *B. fragilis,* and IMP-1 MBLs, respectively [33]. A novel 1β-methylcarbapenem with a trans-3,5-disubstituted pyrrolidinylthio moiety at the C-2 position (**J-111,225**) inhibits the IMP-1 enzyme with a *K*_i_ of 0.18 μM [34]. F. V. Hovel *et al*., [35] also reported penicillin and their rearranged products as potential inhibitors of *B. cereus* MBL.

**Fig. 8:**
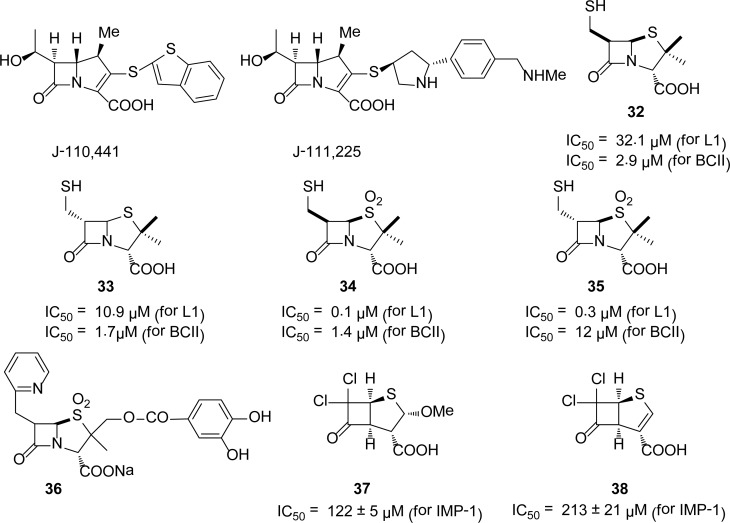
β-Lactam-derived MBL inhibitors

Buynak *et al*., [36] synthesized penicillin-derived inhibitors that simultaneously inhibit both serine and metallo-β-lactamases. The 6-(mercaptomethyl)penicillinates **32–35** (Figure 8) were found as good inhibitors of L1 and BCII MBLs with a *K*_i_ range 0.10–32.1 μM. Tsang *et al*., [37] reported 8-thioxocephalosporins as weak competitive inhibitors (*K*_i_ ∼ 700 μM) of *B.cereus* MBL. Interestingly, the hydrolysis product of thioxocephalosporin, a thioacid, acts as a competitive inhibitor with a *K*_i_ = 96 μM, while the cyclic thioxo-piperazinedione, formed by intramolecular aminolysis of thioxo-cephalexin, inhibits the same enzyme with a *K*_i_ of 29 μM. Badarau [38] and his coworkers also reported that the hydrolysis products of cephalosporins and thiols inhibit the *B.cereus* MBL at the micromolar range. Beharry *et al.,*[39] identified 6-alkylidene-2-substituted penam sulfones as inhibitors of BIa2 with IC_50_ values less than 10 μM. Compound **36** is the representative of this series with the lowest IC_50_ of 1.0 μM. A series of cephalosporin-derived reverse hydroxamates and oximes were prepared and tested against MBLs for inhibitory activity. The reverse hydroxamates were found to inhibit the GIM-1 MBL at the submicromolar level [40]. Cyclobutanone analogues of β-lactams [41] (**37** and **38**) were also reported as the inhibitors of the IMP-1 enzyme.

## Peptides

Sanschagrin *et al*., [42] reported a peptide as an inhibitor for metallo-β-lactamases. Cys-Val-His-Ser-Pro-Asn-Arg-Glu-Cys was identified as a promising inhibitor of the L1 enzyme showing mixed inhibition, *K*_i competitive_ of 16 ± 4 μM and a *K*_i uncompetitive_ of 9 ± 1μM.

**Fig. 9:**
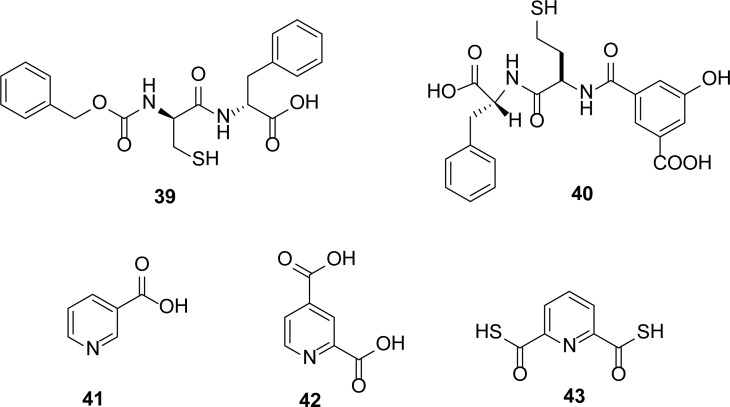
Cysteinyl peptides and pyridine dicarboxylic acid inhibitors of MBLs

Bounaga *et al.,*[23] synthesized several cysteinyl peptides and identified N-carbobenzoxy-D-cysteinyl-D-phenylalanine **39** as the most potent reversible competitive inhibitor of the *B. cereus* MBL with a *K*_i_ value of 3.0 μM. A library of homo-cysteinyl peptides [44] was synthesized and screened for inhibitory activity against L1 metallo-β-lactamase. It was found that homo-cysteinyl peptides are more active than the cysteinyl peptides. The most active compound of the homo-cysteinyl peptides is **40** with a *K*_i_ value of 2.1 nM (Figure 9).

## Pyridine Dicarboxylates

Different pyridine dicarboxylates were tested against different MBLs. 2-picolinic and pyridine-2,4-dicarboxylic acids **41** and **42** (Figure 9) were identified as competitive inhibitors of CphA MBL with *K_i_* values of 5.7 and 4.5 μM, respectively [45]. Roll *et al*., [46] screened natural products, pyridine monothiocaraboxylic acid analogues, for inhibitory activity against MBLs. Among these naturally isolated compounds, dithioacid **43** (Figure 9) was found to be the strongest inhibitor of CcrA from *B. fragilis* and L1 from *S. maltophilia.*

## Natural Products

**Fig. 10:**
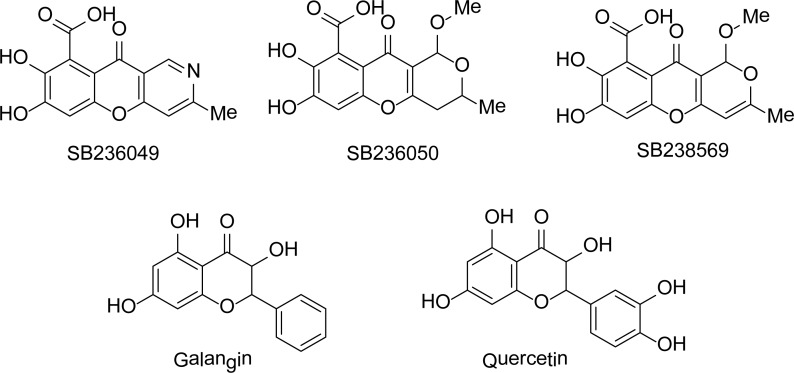
Natural product-based inhibitors of MBLs

Screening of an extract from a strain of *Chaetomium funicola* against *B. cereus* II resulted in the identification of tricyclic natural products (**SB238569**, **SB236050**, and **SB236049**) [47] (Figure 10) as MBL inhibitors. The most active of these natural products was the **SB238569** with *K*_i_ values of 3.4, 17.0, and 79.0 μM for *B. fragilis* CfiA, *P. aeruginosa* IMP-1, and *B. cereus* II MBL, respectively. The flavonoids **galangin** and **quercetin**[48] (Figure 10) were also reported as the inhibitors of MBL from *S. maltophilia*.

## Triazoles and *N*-acylated thiosemicarbazides

**Fig. 11:**
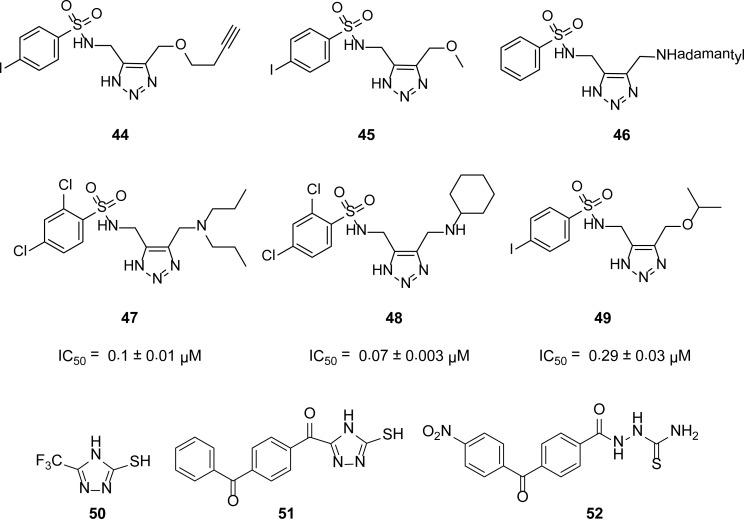
Triazoles and *N*-acylated thiosemicarbazide inhibitors of VIM-2 and IMP-1 MBLs

The VIM-2 enzyme is the most commonly found MBL in clinical isolates worldwide [49–51]. In search of a potent inhibitor of VIM-2, Minond [52] and others screened a library of pharmacologically active compounds and identified two potent and competitive novel sulphonyl-triazoles, **44** (*K*_i_ = 0.41 ± 0.03 μM) and **45** (*K*_i_ = 1.4 ± 0.10 μM), which are inhibitors of VIM-2 MBL. To improve the potency of compound **44**, Weide *et al*., [53] varied the substitutions on the triazole ring and generated the most potent inhibitor **46** of this series with a *K*_i_ of 0.01 ± 0.001 μM. Some other inhibitors **47–49** resulting from this work are given in Figure 11.

Vella *et al*., [2] recently screened a 500 compound Maybridge™ library for several new classes of leading inhibitors against the IMP-1 MBL, and considered the 4-methyl-5-(trifluoromethyl)-4*H*-1,2,4-triazole-3-thiol **50** (*K*_i_ = 0.97 ± 0.60 mM) the most promising for further study. We elaborated this ring system to increase the potency of this compound and identified the mercapto triazole **51** as showing mixed inhibition (*K*_i competitive_ = 75 ± 30 μM and *K*_i uncompetitive_ = 56 ± 10 μM) for the IMP-1 enzyme. We found that the *N*-acylated thiosemicarbazide intermediates in the synthesis of mercapto triazoles are also potent inhibitors of IMP-1 MBL with *K*_i_ values as low as 11μM [54]. *N*-acylated thiosemicarbazide **52** has the maximum inhibitory activity showing mixed inhibition (*K*_i competitive_ = 11 ± 4 μM and *K*_i uncompetitive_ = 20 ± 5 μM) for the IMP-1 enzyme Figure 11.

## Hetrocyclic Thiones and Disulfides

**Fig. 12:**
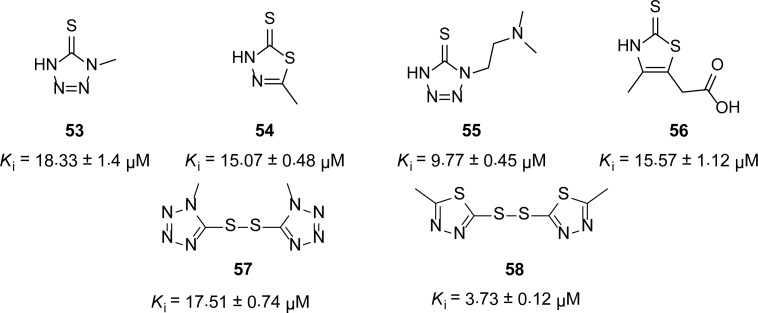
Thiones and disulfides as BcII enzyme inhibitors

Tamilselvi and Mugesh [55] studied the hydrolysis of cephalosporins and found that cephalosporins having heterocyclic -SR side chains are less prone to MBL-mediated hydrolysis. This is partly due to the inhibitory activity of the thione moieties eliminated during hydrolysis. They carried out the enzymatic hydrolysis of oxacillin in the presence of hetrocyclic thiones and disulfides **53–58** (Figure 12) and found that the catalytic activity of the BcII enzyme was significantly reduced by these compounds. However, these inhibitors are not as potent as the aliphatic thiol **9**[12].

## Pyrrole Derivatives

**Fig. 13:**
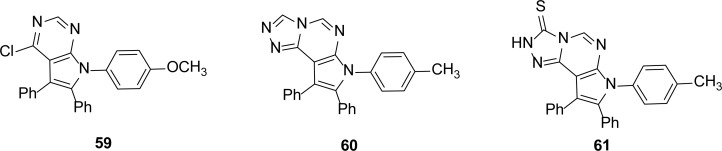
Pyrrole Based inhibitors of the IMP-1 enzyme

Mohamed *et al*., [56] recently synthesized pyrrole derivatives and tested for their inhibitory activity against the IMP-1 enzyme from *P. aeruginosa* and *Klebsiella pneumonia*. They reported some compounds showing micromolar inhibition constants for IMP-1 MBL. Compound **59** showed the maximum inhibition with a *K*_i_ value of 12 ± 4 μM, while compounds **60** and **61** (Figure 13) have *K*_i_ values of 15 ± 5 μM and 18 ± 9 μM, respectively.

## Single Stranded DNAs

Kim [57] and his colleagues identified single-stranded DNAs as rapid, reversible, and non-competitive inhibitors of *Bacillus cereus* 5 / B / 6 MBL, with *K*_i_ and *K*′_i_ values in the nanomolar range. They assayed these ssDNA’s against other zinc-dependent metalloenzymes like porcine carboxypeptidase A, but there was no inhibitory effect on the catalytic activity of these enzymes. Hence, these inhibitors are highly specific to *B. cereus* 5 / B / 6 metallo-β-lactamase. They found 30 residue ssDNA (*K*_i_ = 0.92 nM and *K*′_i_ = 11 nM) and 10 residue ssDNA (*K*_i_ = 0.31 nM and *K*′_i_ = 1.5 nM), the most potent non-competitive inhibitors of *B. cereus* MBL.

## Sulfonic Acid Derivatives

**Fig. 14:**
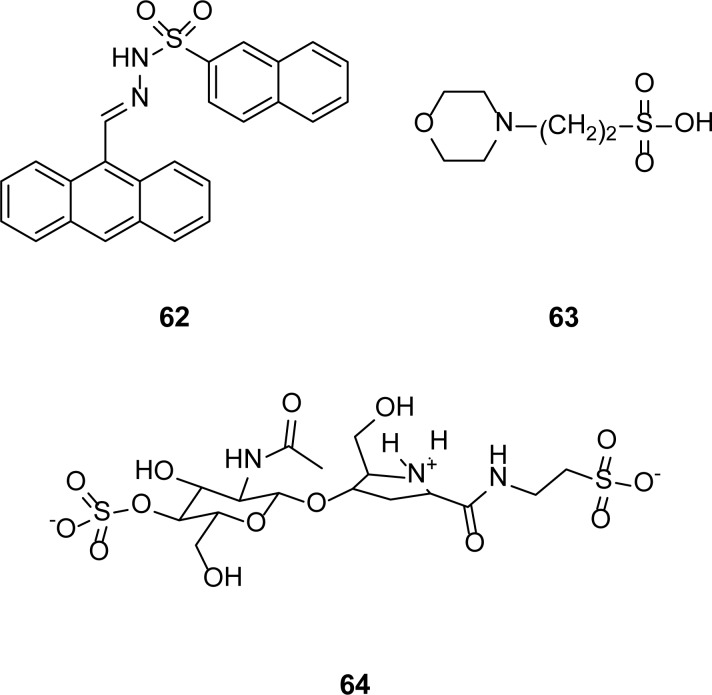
Sulfonated inhibitors of MBLs

Siemann [58] and his coworkers reported N-arylsulfonyl hydrazones as novel inhibitors of the IMP-1 enzyme. The most potent inhibitor of IMP-1 MBL is compound **62** with an IC_50_ of 1.6 μM. 4-Morpholinoethanesulfonic acid **63** has also been reported as a competitive inhibitor of MBL from *B. fragilis* with a *K*_i_ of 23 ± 5 mM [59]. Simm [60] reported bulgecin A **64** as a novel inhibitor of binuclear MBLs. It competitively inhibits (*K*_i_ = 230 ± 10 μM) BcII enzyme in its two-zinc form, but fails to inhibit when the enzyme is in the single-zinc form, while it inhibits (*K*_i_ = 2.5 ± 0.3 μM) the L1 enzyme in a partially competitive mode (Fig. 14).

## Summary

To overcome the problem of increasing resistance of pathogenic bacteria by expressing metallo-β-lactamases to the presently known β-lactam antibacterial agents, several research groups are actively engaged in discovering broad spectrum potent inhibitors of metallo-β-lactamases. Several chemical classes of metallo-β-lactamases have been reported, but there is no such inhibitor to overcome this problem. The challenge for the medicinal chemists and pharmaceutical industries will be to continue to identify such a broad spectrum inhibitor to get rid of this clinical threat. In this review, currently known potent inhibitors of metallo-β-lactamases are presented and we are hopeful that this review could provide a platform for designing a broad spectrum potent inhibitor of all metallo-β-lactamases.

## Authors’ Statement

### Competing Interests

The authors declare no conflict of interests.

## References

[1] Masuda N, Sakagawa E, Ohya S (1995). Outer membrane proteins responsible for multiple drug resistance in *pseudomonas aeruginosa*. Antimicrob Agents Chemother.

[2] Vella P, Hussein WM, Leung EWW, Clayton D, Ollis DL, Mitic N, McGeary PR (2011). The identification of new metallo-β-lactamase inhibitor leads from fragment-based screening. Bioorg Med Chem Lett.

[3] Ambler RP (1980). The structure of beta-lactamases. Philos Trans R Soc London B.

[4] Felici A, Amicosante G, Oratore A, Strom R, Ledent P, Joris B, Fanuel L, Frère JM (1993). An overview of the kinetic parameters of class B beta-lactamases. Biochem J.

[5] Gilpin ML, Fluston M, Payne D, Cramp R, Hood I (1995). Isolation and structure determination of two novel phenazines from a streptomyces with inhibitory activity against metallo-enzymes, including metallo-β-lactamase. J Antibiot.

[6] Payane DJ, Bateson JH, Gasson BC, Proctor D, Khushi T, Farmar TH, Tolson DA, Bell D, Skett PW, Marshall AC, Reid R, Ghosez L, Combret Y, Marchand-Brynaert J (1997). Inhibition of metallo-beta-lactamases by a series of mercaptoacetic acid thiol ester derivatives. Antimicrob Agents Chemother.

[7] Greenlee ML, Laub JB, Balkovec JM, Hammond ML, Hammond GG, Pompliano DL, Epstein-Toney JH (1999). Synthesis and SAR of thioester and thiol inhibitors of IMP-1 Metallo-β-Lactamase. Bioorg Med Chem Lett.

[8] Bounanga S, Laws AP, Galleni M, Page MI (1998). The mechanism of catalysis and the inhibition of the Bacillus cereuszinc-dependent β-lactamase. Biochem J.

[9] Yang KW, Crowder MW (1999). Inhibition Studies on the Metallo-β-lactamase L1 from Stenotrophomonas maltophilia. Arch Biochem Biophys.

[10] Mollard C, Moali C, Papamicael C, Christian Damblon C, Vessilier S, Amicosante G, Schofield CJ, Galleni M, Frere J-M, Roberts GCK (2001). Thiomandelic Acid, a Broad Spectrum Inhibitor of Zinc_β-Lactamases. J Biol Chem.

[11] Kurosaki H, Yamaguchi Y, Higashi T, Soga K, Matsueda S, Yumoto H, Misumi S, Yamagata Y, Arakawa Y, Goto M (2005). Irreversible Inhibition of Metallo-b-lactamase (IMP-1) by 3-(3-Mercaptopropionylsulfanyl)- propionic Acid Pentafluorophenyl Ester. Angew Chem Int Ed.

[12] Siemann S, Clarke AJ, Viswanatha T, Dmitrienko GI (2003). Thiols as Classical and Slow-Binding Inhibitors of IMP-1 and Other Binuclear Metallo-β-lactamases. Biochemistry.

[13] Liénard BMR, Hüting R, Lassaux P, Galleni M, Frère J-M, Schofield CJ (2008). Dynamic Combinatorial Mass Spectrometry Leads to Metallo-β-lactamase Inhibitors. J Med Chem..

[14] Heinz U, Bauer R, Wommer S, Meyer-Klaucke W, Papamichaels C, Bateson J, Adolph HW (2003). Coordination Geometries of Metal Ions in D- or L-Captopril-inhibited Metallo-β-lactamases. J Biol Chem.

[15] Liénard BM, Garau G, Horsfall L, Karsisiotis AI, Damblon C, Lassaux P, Papamicael C, Roberts GC, Galleni M, Dideberg O, Frère JM, Schofield CJ (2008). Structural basis for the broad-spectrum inhibition of metallo-beta-lactamases by thiols. Org Biomol Chem.

[16] Lassaux P, Hamel M, Gulea M, Delbrück H, Mercuri PS, Horsfall L, Dehareng D, Kupper M, Frère JM, Hoffmann K, Galleni M, Bebrone C (2010). Mercaptophosphonate Compounds as Broad-Spectrum Inhibitors of the Metallo-β-lactamases. J Med Chem.

[17] Yamaguchi Y, Jin W, Matsunaga K, Ikemizu S, Yamagata Y, Wachino J, Shibata N, Arakawa Y, Kurosaki H (2007). Crystallographic investigation of the inhibition mode of a VIM-2 metallo-beta-lactamase from Pseudomonas aeruginosa by a mercaptocarboxylate inhibitor. J Med Chem.

[18] Jin W, Arakawa Y, Yasuzawa H, Taki T, Hashiguchi R, Mitsutani K, Shoga A, Yamaguchi Y, Kurosaki H, Shibata N, Ohta M, Goto M (2004). Comparative study of the inhibition of metallo--lactamases (IMP-1 and VIM-2) by thiol compounds that contain a hydrophobic group. Biol Pharm Bull.

[19] Concha NO, Janson CA, Rowling P, Pearson S, Cheever CA, Clarke BP, Lewis C, Galleni M, Frère JM, Payne DJ, Bateson JH, Abdel-Meguid SS (2000). Crystal Structure of the IMP-1 Metallo β-Lactamase from Pseudomonas aeruginosa and Its Complex with a Mercaptocarboxylate Inhibitor: Binding Determinants of a Potent, Broad-Spectrum Inhibitor. Biochemistry.

[20] Gelb MH, Svaren JP, Abeles RH (1985). Fluoro ketone inhibitors of hydrolytic enzymes. Biochemistry.

[21] Brady K, Wei A, Ringe D, Abeles RH (1990). Structure of chymotrypsin-trifluoromethyl ketone inhibitor complexes: comparison of slowly and rapidly equilibrating inhibitors. Biochemistry.

[22] Christianson DW, Lipscomb WN (1986). Complex between carboxypeptidase A and a possible transition-state analog: mechanistic inferences from high-resolution x-ray structures of enzyme-inhibitor complexes. J Am Chem Soc.

[23] Walter MW, Felici A, Galleni M, Soto RP, Adlington RM, Baldwin JE, Frère J-M, Gololobov M, Schofield CJ (1996). Trifluoromethyl alcohol and ketone inhibitors of metallo-β-lactamases. Bioorg Med Chem Lett.

[24] Walter MW, Adlington RM, Baldwin JE, Schofield CJ (1997). Synthesis of metallo-β-lactamase inhibitors. Tetrahedron.

[25] Toney JH, Fitzgerald PM, Grover-Sharma N, Olson SH, May WJ, Sundelof JG, Vanderwall DE, Cleary KA, Grant SK, Wu JK, Kozarich JW, Pompliano DL, Hammond GG (1998). Antibiotic sensitization using biphenyl tetrazoles as potent inhibitors of Bacteroides fragilis metallo-beta-lactamase. Chem Biol.

[26] Toney JH, Cleary KA, Hammond GG, Yuan X, May WJ, Hutchins SM, Ashton WT, Vanderwall DE (1999). Structure-activity relationships of biphenyl tetrazoles as metallo-β-lactamase inhibitors. Bioorg Med Chem Lett.

[27] Toney JH, Hammond GG, Fitzgerald PM, Sharma N, Balkovec JM, Rouen GP, Olson SH, Hammond ML, Greenlee ML, Gao YD (2001). Succinic acids as potent inhibitors of plasmid-borne IMP-1 metallo-beta-lactamase. J Biol Chem.

[28] Moloughney JG, D Thomas J, Toney JH (2005). Novel IMP-1 metallo-β-lactamase inhibitors can reverse meropenem resistance in Escherichia coli expressing IMP-1. FEMS Microbiol Lett.

[29] Olsen L, Jost S, Adolph H-W, Pettersson I, Hemmingsen L, Jørgensen FS (2006). New leads of metallo-β-lactamase inhibitors from structure-based pharmacophore design. Bioorg Med Chem.

[30] Hiraiwa Y, Morinaka A, Fukushima T, Kudo T (2009). Metallo-β-lactamase inhibitory activity of phthalic acid derivatives. Bioorg Med Chem Lett.

[31] Walter MW, Valladares MH, Adlington RM, Amicosante G, Baldwin JE, Frère J-M, Galleni M, Rossolini GR, Schofield CJ (1999). Hydroxamate Inhibitors ofAeromonas hydrophilaAE036 Metallo-B-lactamase. Bioorg Chem.

[32] Liénard BMR, Horsfall LE, Galleni M, Frère J-M, Schofield CJ (2007). Inhibitors of the FEZ-1 metallo-β-lactamase. Bioorg Med Chem Lett.

[33] Nagano R, Adachi Y, Imamura H, Yamada K, Hashizume T, Morishima H (1999). Carbapenem derivatives as potential inhibitors of various β-lactamases, including class B metallo-β-lactamases. Antimicrob Agents Chemother.

[34] Nagano R, Adachi Y, Hashizume T, Morishima H (2000). In vitro antibacterial activity and mechanism of action of J.-111,225, a novel 1β-methylcarbapenem, against transferable IMP-1 metallo-β-lactamase producers. J Antimicrob Chemother.

[35] Hovel FV, Vanwetswinkell S, Marchand-Brynaertt J, FasWez J (1995). Synthesis and rearrangment of potential zinc β-lactamase inhibitors. Tetrahedron Lett.

[36] Buynak JD, Chen H, Vogeti L, Gadhachanda VR, Buchanan CA, Palzkill T, Shaw Rw, Spencer J, Walsh TR (2004). Penicillin-derived inhibitors that simultaneously target both metallo- and serine-β-lactamases. Bioorg Med Chem Lett.

[37] Tsang WY, Dhanda A, Schofield CJ, Frère J-M, Galleni M, Page MI (2004). The inhibition of metallo-β-lactamase by thioxo-cephalosporin derivatives. Bioorg Med Chem Lett.

[38] Badarau A, Llina's A, Laws AP, Damblon C, Page MI (2005). Inhibitors of Metallo-β-lactamase Generated from β-Lactam Antibiotics. Biochemistry.

[39] Beharry Z, Chen H, Gadhachanda VR, Buynak JD, Palzkill T (2004). Evaluation of penicillin-based inhibitors of the class A and B β-lactamases from *Bacillus anthracis*. Biochem Biophys Res Commun..

[40] Ganta SR, Perumal S, Pagadala SR, Samuelsen O, Spencer J, Pratt RF, Buynak JD (2009). Approaches to the simultaneous inactivation of metallo- and serine-beta-lactamases. Bioorg Med Chem Lett.

[41] Johnson JW, Gretes M, Goodfellow VJ, Marrone L, Heynen ML, Strynadka NC, Dmitrienko GI (2010). Cyclobutanone analogues of beta-lactams revisited: insights into conformational requirements for inhibition of serine- and metallo-beta-lactamases. J Am Chem Soc.

[42] Sanschagrin F, Levesque RC (2005). A specific peptide inhibitor of the class B metallo-beta-lactamase L-1 from Stenotrophomonas maltophilia identified using phage display. J Antimicrob Chemother.

[43] Bounaga S, Galleni M, Laws AP, Page MI (2001). Cysteinyl peptide Inhibitors of Bacillus cereus Zinc β-Lactamase. Bioorg Med Chem.

[44] Sun Q, Law A, Crowder MW, Geysen HM (2006). Homo-cysteinyl peptide inhibitors of the L1 metallo-β-lactamase, and SAR as determined by combinatorial library synthesis. Bioorg Med Chem Lett.

[45] Horsfall LE, Garau G, Lie′nard BMR, Dideberg O, Schofield CJ (2007). Competitive inhibitors of the CphA metallo-beta-lactamase from Aeromonas hydrophila. Antimicrob Agents Chemother.

[46] Roll DM, Yang Y, Wildey MJ, Bush K, Lee MD (2010). Inhibition of metallo-beta-lactamases by pyridine monothiocarboxylic acid analogs. J Antibiot.

[47] Payne DJ, Hueso-Rodríguez JA, Boyd H, Concha NO, Janson CA, Gilpin M, Bateson JH, Cheever C, Niconovich NL, Pearson S, Rittenhouse S, Tew D, Díez E, Pérez P, De La Fuente J, Rees M, Rivera-Sagredo A (2002). Identification of a series of tricyclic natural products as potent broad-spectrum inhibitors of metallo-beta-lactamases. Antimicrob Agents Chemother.

[48] Denny BJ, Lambert PA, West PWJ (2002). The flavonoid galangin inhibits the L1 metallo-beta-lactamase from Stenotrophomonas maltophilia. FEMS Microbiol Lett.

[49] Bebrone C (2007). Metallo-beta-lactamases (classification, activity, genetic organization, structure, zinc coordination) and their superfamily. Biochem Pharmacol.

[50] Walsh TR, Tolemman MA, Poirel L, Nordmann P (2005). Metallo-beta-lactamases: the quiet before the storm?. Clin Microb Rev.

[51] Docquier JD, Lamotte-Brasseur J, Galleni M, Amicosante G, Frere JM, Rossolini GM (2003). On functional and structural heterogeneity of VIM-type metallo-beta-lactamases. J Antimicrob Chemother.

[52] Minond D, Saldanha SA, Subramaniam P, Spaargaren M, Spicer T, Fotsing JR, Weide T, Fokin VV, Sharpless KB, Galleni M, Bebrone C, Lassaux P, Hodder P (2009). Inhibitors of VIM-2 by screening pharmacologically active and click-chemistry compound libraries. Bioorg Med Chem.

[53] Weide T, Saldanha SA, Minond D, Spicer TP, Fotsing JR, Spaargaren M, Frère JM, Bebrone C, Sharpless KB, Hodder PS, Fokin VV (2010). NH-1,2,3-Triazole-based Inhibitors of the VIM-2 Metallo-β-Lactamase: Synthesis and Structure-Activity Studies. ACS Med Chem Lett.

[54] Faridoon, Hussein WM, Vella P, Islam NU, Ollis DL, Schenk G, McGeary RP (2012). 3-Mercapto-1,2,4-triazoles and N-acylated thiosemicarbazides as metallo-β-lactamase inhibitors. Bioorg Med Chem Lett.

[55] Tamilselvi A, Mugesh G (2011). Metallo-β-lactamase-Catalyzed Hydrolysis of Cephalosporins: Some Mechanistic Insights into the Effect of Heterocyclic Thiones on Enzyme Activity. Inorg Chem.

[56] Mohamed MS, Hussein WM, McGeary RP, Vella P, Schenk G, Abd El-hameed RH (2011). Synthesis and kinetic testing of new inhibitors for a metallo-β-lactamase from Klebsiella pneumonia and Pseudomonas aeruginosa. Eur J Med Chem.

[57] Kim S-K, Sims CL, Wozniak SE, Drude SH, Whitson D, Shaw RW (2009). Antibiotic resistance in bacteria: novel metalloenzyme inhibitors. Chem Biol Drug Des.

[58] Siemann S, Evanoff DP, Marrone L, Clarke AJ, Viswanatha T, Dmitrienko GI (2002). N-arylsulfonyl hydrazones as inhibitors of IMP-1 metallo-beta-lactamase. Antimicrob Agents Chemother.

[59] Fitzgerald PMD, Wu JK, Toney JH (1998). Unanticipated inhibition of the metallo-beta-lactamase from Bacteroides fragilis by 4-morpholineethanesulfonic acid (MES): a crystallographic study at 1.85-A resolution. Biochemistry.

[60] Simm AM, Loveridge EJ, Crosby J, Avison MB, Walsh TR, Bennett PM (2005). Bulgecin A: a novel inhibitor of binuclear metallo-beta-lactamases. Biochem J.

